# Multiple Site Dissimilarities of Herbaceous Species Due to Coal Fly Ash Dumping Based Soil Heavy Metal Toxication

**DOI:** 10.3390/toxics11020090

**Published:** 2023-01-18

**Authors:** Deepak Kumar Singh, Anushka Singh, Amel Gacem, Shruti Kashyap, Virendra Kumar Yadav, Krishna Kumar Yadav, Hany S. Hussein, Neeraj Kumar Shukla, Amnah Mohammed Alsuhaibani, Magda H. Abdellattif, Chaigoo Lee, Wonjae Lee, Timsi Modi, Byong-Hun Jeon

**Affiliations:** 1 Department of Botany, Guru Ghasidas Vishwavidyalaya, Bilaspur 495001, Chhattisgarh, India; 2 Department of Physics, Faculty of Sciences, University 20 Août 1955, Skikda 21000, Algeria; 3 Department of Biosciences, School of Liberal Arts & Sciences, Mody University of Science and Technology, Lakshmangarh, Sikar 332311, Rajasthan, India; 4 Faculty of Science and Technology, Madhyanchal Professional University, Ratibad 462044, Bhopal, India; 5 Electrical Engineering Department, Faculty of Engineering, King Khalid University, Abha 61411, Saudi Arabia; 6 Electrical Engineering Department, Faculty of Engineering, Aswan University, Aswan 81528, Egypt; 7 Electrical Engineering Department, College of Engineering, King Khalid University, Abha 61421, Saudi Arabia; 8 Department of Physical Sport Science, College of Education, Princess Nourah bint Abdulrahman University, P.O. Box 84428, Riyadh 11671, Saudi Arabia; 9 Department of Chemistry, College of Science, Taif University, Al-Haweiah, P.O. Box 11099, Taif 21944, Saudi Arabia; 10 Golden Engineering Co., Ltd., Seoul 05836, Republic of Korea; 11 Department of Environment Sciences, School of Sciences, P P Savani University, Surat 394125, Gujarat, India; 12 Department of Earth Resources & Environmental Engineering, Hanyang University, 222-Wangsimni-ro, Seongdong-gu, Seoul 04763, Republic of Korea

**Keywords:** heavy metals, species diversity, species count, edaphic factors

## Abstract

The present study analyzes the determinants and patterns of the regional, local, and differential plant diversity of two different sites with similar climatic but varied edaphic factors. This research was undertaken to study the plant diversity and population structure as a consequence of variation in the soil quality between two biotopes: Guru Ghasidas Vishwavidyalaya in Koni (site-I) and National Thermal Power Corporation in Sipat (site-II). The soil of site-I was found to be fertile and showed rich vegetation. On the other hand, the soil of site II was found to be contaminated with heavy metals, which impacts the flora of the region. The ecology of both sites was studied, and their quantitative and qualitative aspects were compared and contrasted. The abundance, density, and richness of the plants in site II were fairly lower than in site-I, which was confirmed by utilizing Simpson’s and Shannon’s diversity indices. Many of the species collected from site II were heavy metal accumulators and could also serve as indicators of heavy metal toxicity.

## 1. Introduction

The unusual distribution and diversity of plants have always been a subject of interest among researchers [1]. A fair number of equilibrium and non-equilibrium theories have been proposed to explain different diversities, such as spatial variation in resource availability, fire, grazing, climate, etc. The most common perspective on plant ecology is that a region’s climate is the only deterministic factor of plant diversity [2]. However, finer environmental features, such as edaphic factors, have a significant role in shaping the flora of a place [3]. The question of which factor determines the occurrence and distribution of species certainly is still unanswered for much of the world. Biodiversity is one of the important factors of a healthy ecosystem, and it must be maintained. However, nowadays, biodiversity is being lost due to several factors, including climate change, invasive species, the over-exploitation of natural resources, pollution, and urbanization [4].

From various pieces in the literature, it is evidenced that the richness, abundance, and distribution of plant species in an area can be attributed to climatic, geographic, edaphic, and historic factors and perturbations. In areas with relatively similar climatic conditions and environmental factors, the edaphic factors are solely responsible for contrasting patterns of hyper-diversification among plant species. Moreover, some of the investigators concluded that all these above-mentioned factors could be measured and studied individually, but their interaction with each other and with organisms must always be considered together.

Out of these factors, the present study focuses on the edaphic factors that lead to the presented distribution. From the literature, it is well proven that the soil characteristics, for instance, soil salinity; surface soil acidity; soil electrical conductivity; exchangeable magnesium, calcium, and potassium; soil organic carbon content; heavy metals, moisture content, porosity, etc. impact the distribution of plants within the region. Soil is regarded as saline if it contains salt in a quantity that hampers the crops. It is considered a major limiting factor to cropping [5]. The soil acidity majorly restricts the minor and major mineral nutrient uptake of plants. The high acidity of soil with a low calcium content and sometimes the toxic levels of soluble or exchangeable aluminum metal severely impair plant root development in these soils [6]. The optimum level of exchangeable soil magnesium levels ranges from 25 to 180 ppm. If the exchangeable Mg content is high in the soil, it also causes hypomagnesemia in the ruminants which consume it.

Among all the above-mentioned soil characteristics which affect plant growth and variation is heavy metal. From examples in the literature, it is evident that the variety of plants that grow in any particular kind of soil largely differs from the variety of plants that grow in the same kind of soil but with heavy metal contamination. Urbanization and industrialization have both drastically increased the concentration of heavy metals in the biosphere, which earlier used to be restricted to the area with rocks and in places that have lots of ores. Different plant species show different levels of tolerance regarding the presence of heavy metals in the soil. Some of the investigators have reported that heavy metals, copper, zinc, arsenic, cadmium, lead, iron, etc., are the most widely occurring metals in industrialized areas. Different heavy metals have different impacts on the plant; for instance, copper is considered essential heavy metal for some higher plants and for algae, as it seems to assist in photosynthesis [7].

There are several sources of heavy metals, such as industries, coal fly ash (CFA), and other waste, such as red mud. CFA are micron-sized, heterogeneous, glassy, spherical byproducts that are generated at thermal power plants (TPPs) during the production of electricity from pulverized coal [8,9,10]. Since coal is a geological material that contains several elements present in the soil, the CFA also has several beneficial and toxic elements. It has mainly three major oxides: silicon dioxide, alumina, and ferrous. CFA has several non-heavy metals, including Fe, Si, Ca, Zn, Mg, Br, C, etc., which is considered an essential nutrient for the plant. In addition to this, it has several toxic heavy metals such as Zinc (Zn), Nickel (Ni), Lead (Pb), Mercury (Hg), Cadmium, Arsenic (As), Sr, etc. [11,12]. Yadav and Fulekar 2020, and Yadav et al., have also shown the presence of such toxic heavy metals in the CFA from Gandhinagar, Gujarat TPPs [13,14,15,16,17,18].

CFA is generally dumped in the CFA ponds near the vicinity of the TPPs. During the rainy season and by coming into contact with water, the heavy metals may leach out from the CFA and may reach aquatic systems and agricultural lands [19]. So, these heavy metals may combine with water and soil and heavily impact the soil-plant processes. In addition to this, the ultrafine particles from TPPs may remain suspended in the air for a prolonged period which could cause adverse effects on living beings.

So, while selecting this area for study, several factors were considered. This area (Sipat, Bilaspur, Chhattisgarh) has one of the largest TPPs (super TPPs) owned by the National Thermal Power Corporation (NTPC). This superthermal power plant (STPP) is the source of power not only for the state of Chhattisgarh but also for six neighboring states.

Several investigators have carried out investigations on the coal mines, CFA, soil, and plant species of this area, mainly the Korba region of Chhattisgarh; for instance, Singh et al., 2022, reported the isolation of a few chemolithotrophic siderophore-producing bacteria from the coal mines from this region [20]. In one more investigation carried out by Shukla (2018), the author reported the presence of about 30 different types of ethnomedical plant species from this region. These plants were mainly used by the Korba tribes for the healing of several diseases, and these plants were considered to have great socio-economic importance [21]. In one investigation carried out by Bhaskar and Dixit 2015, on the water quality of the Hasdeo river, from the Korba region. The authors reported the presence of numerous pollutants above the prescribed limit in the water samples. Among the heavy metals, Mn, Pb, and Fe were much higher. The Fe was between 13 and 19-fold higher than the normal limit. The authors concluded that this water was not fit for drinking and could only be used for irrigation purposes [22].

In the current research work, the authors tried to present the variation in types of herbaceous species of plants among areas with heavy metal-contaminated soil and areas with no such contamination. The data obtained from the field were used to analyze the quantitative and qualitative aspects of the plant population. Further, the collected data were used to measure frequency, density, abundance, relative frequency, relative density, relative abundance, etc. which provided a quantitative map of the population, whereas qualities of the population, such as richness, evenness, and diversity, can be estimated by plotting graphs including rarefaction/accumulation curves and rank abundance curves [23]. Further, indices similar to Simpson’s and Shannon’s Index were calculated to validate the richness and evenness, respectively. Finally, all these features were combined together to provide the characteristics of a population, which helped in the measurement of the biodiversity of that place.

From prior studies, it was found that a stronger study was required in this area, especially the effect of bad air, water, and soil quality on the herbaceous species of plants. The objectives of the current study are thus able to compare and contrast the diversity of two regions with different soil characteristics. Another objective is to provide the characteristics of the plant population of the study area by relevant calculations. Yet, another objective is to highlight the role of edaphic factors in the biodiversity of any region. The final objective is to list the ecological importance of phytodiversity in an area with heavy metal stress.

## 2. Materials and Methods

### 2.1. Materials

HNO_3_ (SRL, Delhi, India), H_2_SO_4_ (Renkem, New Delhi, India), HClO_4_(SRL, New Delhi, India), and Whatman filter paper 42 (Axiva, New Delhi, India).

### 2.2. Study Sites

**Figure 1:**
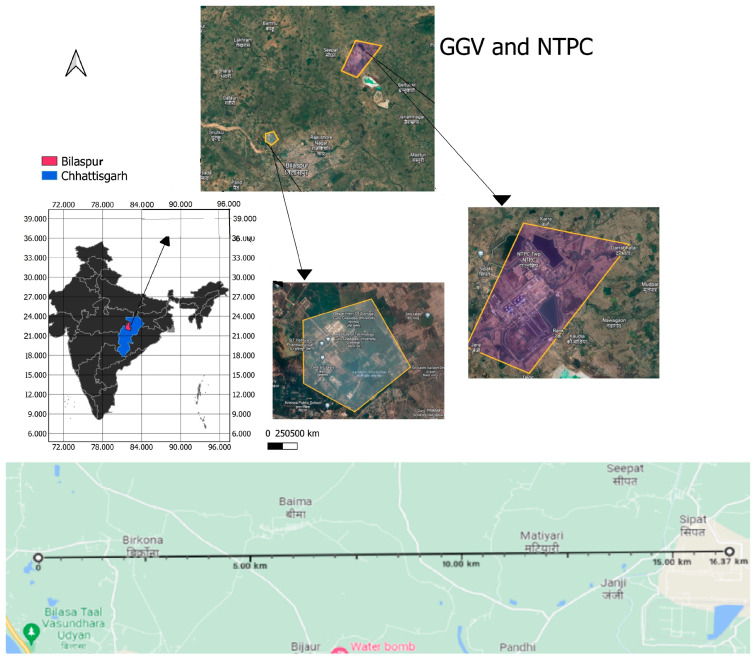
Study site location. GIS, 2022.

The present study took place in two sites: Guru Ghasidas Vishwavidyalaya (GGV) Campus, Koni, Bilaspur, and National Thermal Power Corporation, Sipat Bilaspur. GGV (site-I) lies at 22.1293° N, 82.1360° E, and NTPC (site-II), as shown in Figure 1, lies at 22.1377° N, 82.2907° E (shown in Figure 1). These sites are located 17 kilometers apart within the same Bilaspur district. The annual precipitation of the district is about 58 cm. December is the driest month of all. The data collection for the present study was performed in the month of April when the temperature ranged between 33 and 46 °C. Both sites have similar climatic conditions but very different soil characteristics.

### 2.3. Sampling of the Soil

Both study sites were divided into several homogenous units, and samples were collected randomly from three distant units. After removing the surface litter, a V-shaped cut at a depth of 15 cm was made on the ground from where the soil was collected using a spade. The same procedure was performed for both study sites. The samples were collected in polythene bags and labeled with the collector’s name, place, and time of collection.

### 2.4. Data Collection

The data were collected through regular field visits for two weeks, from 11 to 26 April 2022. The sampling of plant species was performed by a random sampling quadrat method for both site-I and site II. The quadrats laid were 1 × 1 m^2^ in size. There were 40 quadrats laid in site II and 40 quadrats in site-I. Pictures and samples of all plant species were collected and sent for identification. The documentation of each and every species was important because very little information was available about the population dynamics and diversity of South Asia. The species were sorted on the basis of their frequency, and ranks were allotted to each in terms of their abundance. The frequency, abundance, density, relative frequency, relative abundance, and relative density was calculated and tabulated (Ara, 2020). The Importance Value Index for each of these was also calculated. After the segregation of the species on the basis of their abundance, they were allotted respective ranks, and a rank-abundance graph was plotted for both places. The physical properties of the collected soil samples from both sites were determined. The specific gravity of soil was calculated in comparison to that of water. The texture was analyzed by particle size analysis of the soil at 15 cm below the surface.

### 2.5. Data Analyzation

(1) The diversity of species from site II was contrasted with the one from site-I. The frequency, abundance, and density of species that were found in both places were also compared and contrasted. The species found exclusively on-site II and site-I were analyzed and the reasons for such diversity were critically investigated, as shown in Table 1 and Table 2.
(2) The pH of the soil from both the study sites was tested separately for their physical properties and the concentration of metals present in both of them. The pH was measured using a pH meter (Analab, Gujarat, India) which is precise up to ±0.1 pH unit that is accepted to be adequate for field work [24,25]. The electrical conductivity (Analab, Gujarat, India) was measured in a conductivity cell by measuring the electrical resistance of 1:1 soil: water suspension with two electrodes placed 0.01 m apart [26]. The measurement was taken in deciSiemens per meter(dS/m). The organic carbon content of the soil was calculated in percentage by utilizing Walkley and Black’s colorimetric method [27].
(3) The sample soil from both of the sites was collected and labeled separately. Both of the samples weighing 0.5 g each were digested with 15 mL HNO_3_, H_2_SO_4_, and HClO_4_ in a ratio of 5:1:1 by a hot plate open vessel approach at 80 °C until a transparent solution was obtained. The solution was filtered through Whatman Grade 42 quantitative papers and was diluted to 50 mL. The concentration of heavy metals in each of the samples was then determined with an atomic absorption spectrophotometer (AAS) [Model: ICE3300, Make: Thermo Scientific], USA [28].

### 2.6. Calculation

The Simpson and Shannon indices were calculated for both the biotopes. From Site-I, the highest number of plants for the species encountered was *Cynodon dactylon* L., *Parthenium hysterophorus, Digitaria sanguinalis, Oxalis corniculate,* and *Evolvulus nummularius* at 548, 202, 108, 104, and 97, respectively, as the total number of individuals found. From site II, the number of plants for *Cynodon dactylon* was 158, and *Parthenium hysterophorus* was 164. The above-listed species are the most abundant ones. By comparing the two sites, the authors observed that the abundance of the same species in the two sites evinced anomaly as their abundances were greater in site I than in site II.

(1) $$\mathrm{Frequency}=\frac{numberofquadrants\in whichspeciesoccurred}{totalnumberofquadrantsstudied}\times 100\;$$

(2) $$\mathrm{Abundance}=\frac{totalnumberofindividualofspecies}{No.ofquadrateperunits\in whichtheyoccur}\times 100$$

(3) $$\mathrm{Density}=\frac{Totalno.ofindividualofthespecies}{No.ofquadratperunitsstudied}\times 100$$

(4) $$\mathrm{Relative}\;\mathrm{frequency}=\frac{Frequencyofindividualspecies}{Totalfrequencyofallspecies}\times 100$$

(5) $$\mathrm{Relative}\;\mathrm{density}=\frac{Densityofindividualspecies}{Totaldensityofallspecies}\times 100$$

(6) $$\mathrm{Relative}\;\mathrm{abundance}=\frac{Abundanceofindividualspecies}{Totalabundanceofallspecies}\times 100$$

IVI=RF+RA+RD

## 3. Results

There were 40 quadrats laid on the GGV campus (Site-I). The campus is relatively greener and hosts 58 herbaceous species belonging to 23 different families. The most frequent species of the biotope are *Cynodon dactylon* and *Parthenium hysterophorus,* with frequencies of 46.6 and 68.8, respectively. Their frequencies are 79 and 86 percent higher than the average frequencies for all the herbaceous plants (site-I). The reason for such a distinction is the invasive nature of these species. The least occurring species is *Malvastrum coromandelianum*. Of all the families, Poaceae and Asteraceae are the most frequent, with 22 and 17 species recorded within the campus, respectively. Simpson’s index for this distribution is 0.959, and Shannon’s index is 1.44.

Out of the 40 quadrats laid in site II, 28 species belonged to 17 families. The abundance of these species was less than the species from the site I. The most frequent family in this region is Asteraceae and Amaranthaceae. Other families like Cleomaceae, Portulacaceae, Onagraceae, and Primulaceae were only recorded from site II. The most abundant species were *Cynodon dactylon*, *Cyperus rotundus,* and *Parthenium hysterophorus,* with an importance value index of 22.23, 34.46, and 35.71, respectively.

**Figure 2:**
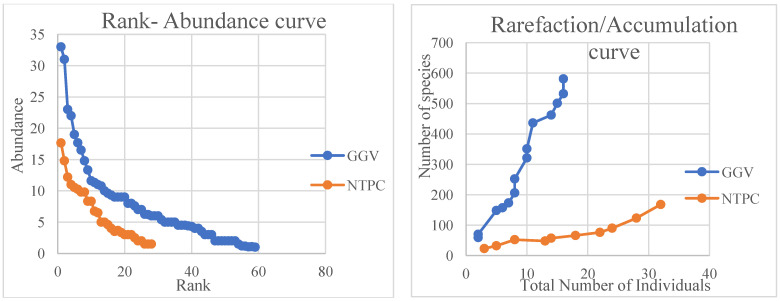
Rank–abundance curve and rarefaction curve of GGV(Site-I) and NTPC(Site-II).

The species were allotted ranks with respect to their abundances, and a rank–abundance curve was plotted. The number of individuals increased with the number of species encountered and is plotted as a rarefaction curve, as shown in Figure 2.

These trends of variation in species diversity can be explained by the analysis of soil from both places, shown in Table 3.

The above table shows concentrations of heavy metals in the soil of GGV(Site-I) vs. NTPC (Site-II). The electrical conductivity of both kinds of soil falls at a normal range and is non-saline. The organic carbon in the case of soil from site II is significantly lower than normal; however, the soil from site-I has sufficient organic carbon. The specific gravity of contaminated soil is lower than that of unpolluted soil. However, there is not much difference in the particulate nature of the soil at a depth of 15 cm from the surface. Yet, at the molecular level, there was a huge difference between both. The concentration of nitrogen and phosphorus is significantly low in the soil from site II. However, micronutrients such as iron (Fe), manganese (Mn), copper (Cu), and boron (B) are at higher-than-normal levels. Iron is the most abundant micronutrient in the soil, followed by manganese. The soil from site II also has a sufficient lead (Pb) concentration. In contrast to that, Pb in the soil of site I is non-traceable. These antagonistic soil properties have resulted in a contrasting variety of plants in two regions with similar climatic conditions.

## 4. Discussion

The research was conducted on two sites that were exposed to similar climatic conditions, water availability, and soil type. The only difference between both sites is that the soil from site II was contaminated with heavy metals. The plants which were wild and native to both sites were studied, and the ones that were introduced artificially were excluded from the study. The only difference in contamination resulted in many anomalies between the biodiversity of both the sites observed in the richness, type, and abundance of the species encountered. Site-I was found to be richer than site-II. The reason for this distinction is the soil pollution caused by the deposition of CFA in the soil coming from the STPPs [29,30,31,32,33,34,35,36]. The CFA is stored in the fly ash ponds [37] near the vicinity of TPPs, from where CFA is released in the form of a slurry mixed with water. Gradually, the slurry is dried and transported through the wind to settle in water bodies and in soil, causing much pollution [38]. CFA is harmful to humans and is a very prominent disease-causing agent in areas near power plants [39]. Before 2017 the level of heavy metals in the soil was much higher than today. Utilizing the ash in making fly ash brick (FAB) and in road making has decreased the level of CFA deposits in dams [40]. However, the soil still remains polluted due to the smoke coming out of chimneys. The air quality index (AQI) of NTPC is very poor as the particulate matter, i.e., PM_2.5_ was found to be 208. The presence of such a high particulate matter in the NTPC region has directly impacted the richness and diversity of the region. There are several species found which serve as good indicators of pollution and heavy metal concentration. On the other hand, the Guru Ghasidas Vishwavidyalaya campus is a place with the least pollution. The types of plants found in both places are different. Even the plants that are common in both sites evince clear morphological differences, e.g., the size and number of stomatal pores on the ventral and dorsal sides of leaves.

These heavy metals were also detected in plants and their parts. The flowering plants can accumulate these heavy metals in their nectar which affects the pollinators and nectar robbers and also alters the plant’s reproductive ability [9,10,11,41,42,43,44,45]. The kind of plant species recorded from both places also differs significantly.

On comparing site-I with site II, it became evident that the latter had higher levels of iron, manganese, boron, and copper. These micronutrients in their optimum quantity are very important for the plant’s growth and development, but higher levels than required can negatively impact the plant. Iron is the most abundant heavy metal found in the soil of site II. Iron toxicity can cause irreversible damage to cells, membranes, DNA, and RNA. Manganese and Iron are competitive for absorption as an abundance of one causes the depletion of the other. Manganese toxicity can easily be spotted as the leaves become ‘crinkled’. A high level of copper in the soil can cause reduced root development in plants.

The abundance of plants from site II is much less than from site-I. The Simpson’s index calculated for Site-I is 0.959, whereas, for Site-II, it is 0.088. This shows that site-I is more diverse than site II. Shannon’s index for site-I is 1.44, and for site-II, it is 1.09, which affirms that Site-I has a richer diversity than site-II. The soil organic carbon of site II was found to be 0.45%, which is 95.9 percent less than the soil organic carbon of site-I. This abnormality could be due to the competition between the plants for available nutrients in the soil. Additionally, the metal toxicity in plants reduces their reproductive fitness, which lowers the chances of their propagation. The survival of plants in heavy metal toxicity areas is also very low. Out of the total number of plants recorded from site II, 53% of plants had the ability to accumulate heavy metals or survive heavy metal toxicity. The plants *Cynodon dactylon* and *Parthenium hysterophorus* are abundant in both biotopes because of their invasive nature [46]. *P. hysterophorus* is known to remove heavy metals from the habitat [47]. There are several species that are found only on-site II and not on site-I. These are, *Portulaca oleracea*, *Hyptis suaveolens*, *Grangea maderaspatana*, *Sphaeranthus indicus*, *Peperomia pellucida*, *Setaria verticillata*, *Hygrophylla auriculata*, *Tephrosia* sp., *Ocimum* sp., *Clarkia amoena*, *Lysimachia nummularia*, *Dichondra argentea*, *Jatropha curcas*, and *Celosia*. Unsurprisingly, all of these species from site II have specific properties and heavy metal accumulation abilities that the plants from site-I lack.

The plant *Portulaca oleracea* has the potential to accumulate lead [48,49]. *Hyptis suaveolens* is now used for the Phytoremediation of polluted soil due to its property of accumulating heavy metals in its stem and leaves. The seeds of this plant have the potential for Arsenic bioabsorption. The most abundant heavy metal accumulator of site-II is *Grangea maderaspatana.* This is a plant from the family Asteraceae, which has the potential to absorb lead and cadmium from the soil. *Sphaeranthus indicus* is known for the phytoremediation of copper-tinted soil [50]. Lead contamination in soil is the most concerning and highly encountered phenomenon. Plants such as *Peperomia pellucida* absorb lead in their roots which is indicated by their BCF value [51]. The plants that accumulate lead show symptoms, including chlorosis, wilting, and necrosis. Similarly, the bioaccumulation of nickel is performed by *Tephrosia* sp.

*Dichondra argentea* species from the family Convolvulaceae has the highest Phytobarriers index of all the species encountered. It has the unique ability to absorb the potentially toxic particles in its leaves from the air and reduce their dispersion [52]. This property is very relevant from ecological and environmental perspectives. *Jatropha curcas* is now used for the phytoremediation of soil contaminated with Cd, Ni, Cr, and Zn. Apart from the heavy metal accumulation capability of these plants, they also have pharmacological importance as well. Thus, the species that are present in site II and site-I differ on the grounds of their ability to adapt to adverse soil conditions.

## 5. Conclusions

Biodiversity is an important parameter for a sustainable environment. The environment and climate change, including edaphic factors, have an impact on the growth pattern and variation in plant species. The fly ash produced by thermal power plants has toxic heavy metals which leach into the nearby agricultural field and soil and change the chemical constituents of the soil. This fly ash contains heavy metals that degrade the quality of the soil. The present study on the plant species in the nearby area of fly ash dumping sites is evident when edaphic factors, such as soil salinity and heavy metal concentrations, impact the diversity of plants. In fertile soil conditions, the species count, abundance, density, and richness are significantly higher than the polluted soil. The contaminated soil has a significantly lower level of organic carbon than the uncontaminated soil. The percentage composition of essential soil minerals such as Nitrogen, Phosphorus, Potassium, and Zinc is also lower in contaminated soil. The presence of lead is the highest among all the heavy metals found in the soil.

The presence of heavy metals in soil has an adverse effect on the growth and development of the plant. It also decreases the chances of a healthy crop and negatively impacts the agroecosystem. The plants that are common in both the study sites have morphological differences such as seed morphology, the height of the plants, and the size of the stomata, etc. The soil pollution due to the fly ash deposition also poses a threat to the health of crops.

The abundance, frequency, density, etc., quantities were utilized to highlight the change in the population of different species due to the edaphic factors. Further, it was found that plant species from specific areas had remarkable potential for phytoremediation and occurred naturally in polluted soil. While the same plant species grown on normal fertile soil did not exhibit such properties as shown by the heavy metal-contaminated soil.

## Figures and Tables

**Table 1:** Calculation for herbaceous species from the GGV campus (Site-I).

S.no	Species Name	Frequency	Density	Abundance	Relative Frequency	Relative Density	Relative Abundance	IVI
1	*Poa annua*	13.3	1.77	13.33	2.37	5.16	2.91	10.4
2	*Digitaria cilaris*	2.22	0.733	33	0.396	0.73	7.22	8.346
3	*Alternanthera sessilis*	22.2	0.97	4.4	3.96	2.11	0.96	7.03
4	*Oxalis corniculata*	15.55	2.31	14.8	2.78	5.03	3.23	11.04
5	*Cyperus rotundus*	2.22	0.11	5	0.39	0.23	1.09	1.71
6	*Parthenium hysterophorus*	46.6	4.48	9.61	8.33	9.76	2.1	20.19
7	*Desmodium triflorum*	13.3	2.17	16.5	2.37	4.73	3.61	10.71
8	*Cynodon dactylon* L.	68.8	12.17	17.67	12.3	26.53	3.86	42.69
9	*Digitaria sanguinalis*	26.6	2.4	9	4.75	5.23	1.96	11.94
10	*Richardia brasiliensis*	4.44	0.088	2	0.79	0.191	0.43	1.411
11	*Chrozophora plicata*	2.22	0.11	5	0.396	0.23	1.093	1.719
12	*Acmella uliginosa*	8.88	0.177	2	1.58	0.38	0.43	2.39
13	*Ammannia baccifera*	2.22	0.022	1	0.396	0.047	0.21	0.653
14	*Menthera piperita*	6.66	0.622	9.3	1.19	1.35	2.03	4.57
15	*Erigeron floribundus`*	20	1.24	6.2	3.57	2.7	1.35	7.62
16	*Malvastrum coromandelianum*	2.22	0.06	3	0.396	0.13	0.65	1.176
17	*Cyanthillium cinereum*	15.55	0.71	4.5	2.78	1.54	0.98	5.3
18	*Evolvulus nummularius* L.	20	2.15	10.77	3.57	4.68	2.35	10.6
19	*Alternanthera paronychioides*	17.77	1.11	6.25	3.17	2.41	1.36	6.94
20	*Chloris virgata*	11.11	1.28	11.6	1.98	2.79	2.46	7.23
21	*Paspallum setaceum*	2.22	0.2	9	0.396	0.43	1.96	2.786
22	*Paspallum conjugetum*	2.22	0.24	11	0.39	0.52	2.4	3.31
23	*Carex blanda*	2.22	0.13	6	0.39	0.28	1.31	1.98
24	*Eriophyes cynodoniensis*	2.22	0.51	23	0.39	1.11	5.03	6.53
25	*Elusine indica*	2.22	0.08	4	0.39	0.17	0.87	1.43
26	*Blumea lacera* L.	6.66	0.26	4	1.19	0.56	0.87	2.62
27	*Lolium perenne*	2.22	0.04	2	0.39	0.08	0.43	0.9
28	*Paspallum notanum*	2.22	0.42	19	0.39	0.91	4.15	5.45
29	*Senecio vulgaris*	2.22	0.04	2	0.39	0.08	0.43	0.9
30	*Gnaphalium polycaulon*	4.44	0.13	3	0.79	0.28	0.65	1.72
31	*Eragrostis amabilis*	4.44	0.26	6	0.799	0.56	1.31	2.669
32	*Eragrostis hirta*	6.66	0.28	4.3	1.19	0.61	0.94	2.74
33	*Cyperus alulatus*	2.22	0.17	8	0.39	0.37	1.75	2.51
34	*Zoyria matrella*	2.22	0.68	31	0.39	1.48	6.78	8.65
35	*Sporobolus indicus*	2.22	0.22	10	0.39	0.47	2.18	3.04
36	*Brachiaria reptans*	6.66	0.511	7.6	1.19	1.11	1.66	3.96
37	*Cassia tora*	11.11	0.822	5.4	1.98	1.787	1.81	5.57
38	*Chamaesyce uspidat*	4.44	0.4	9	0.799	0.87	1.96	3.62
39	*Chromoleana odorata*	4.44	0.2	4.5	0.799	0.43	0.98	2.20
40	*Euphorbia prostrata*	2.22	0.11	5	0.39	0.24	1.09	1.72
41	*Hieracium*	26.6	0.288	1.083	4.75	0.611	0.23	5.59
42	*Laggera aurita*	42.22	0.44	1.05	7.54	0.96	0.22	8.72
43	*Lapidegathis uspidate Nees.*	2.22	0.044	2	0.39	0.096	0.43	0.91
44	*Oplismenus hirtellus*	13.33	0.15	1.16	2.38	0.32	0.25	2.95
45	*Phyllanthus maderaspatensis*	4.44	0.066	1.5	0.79	0.144	0.32	1.25
46	*Sarghastrum nutans*	11.11	0.133	1.2	1.98	0.29	0.26	2.53
47	*Tridax procumbens*	13.33	0.93	7	2.38	2.03	1.53	5.94
48	*Choprosoma abconia*	2.22	0.044	2	0.39	0.096	0.43	0.91
49	*Chrozophora tinctoria*	2.22	0.17	8	0.39	0.37	1.75	2.51
50	*Eleusine indica* L.	8.8	0.4	4.5	1.57	0.87	0.98	3.42
51	*Indigo feratinctoria* L.	2.22	0.066	3	0.39	0.144	0.65	1.18
52	*Malvestrum coromandelianum*	4.44	0.266	6	0.79	0.56	1.31	2.66
53	*Medicago*	2.22	0.15	7	0.39	0.32	1.53	2.24
54	*Panicum brevifolium* L.	2.22	0.11	5	0.39	0.24	1.093	1.72
55	*Platylobium rotundrum*	6.66	0.75	11.33	1.19	1.63	2.47	5.29
56	*Rungia pectinata*	8.8	0.8	9	1.57	1.74	1.96	5.27
57	*Scenecio vulgaris*	2.22	0.044	2	0.39	0.096	0.43	0.91
58	*Sida cardifolia*	4.44	0.155	3.5	0.79	0.33	0.76	1.88
59	*Urochloa platyphylla*	2.22	0.488	22	0.39	1.04	4.81	6.24
	Total-	559.28			99.737	100.192	100.256	
	Mean and Standard deviation-	9.47 ± 149.21	0.77 ± 2.85	7.74 ± 47.79	1.71 ± 4.80	1.72 ± 13.93	1.72 ± 2.28	

**Table 2:** Calculation for herbaceous species from NTPC, Sipat (Site II).

S.no.	Species Name	Frequency	Density	Abundance	Relative Frequency	Relative Density	Relative Abundance	IVI
1	*Cynodon dactylon*	37.5	3.95	10.53	11.36	17.09	6.01	34.46
2	*Alternanthera sessilis*	12.5	1.22	9.8	3.78	5.28	5.59	14.65
3	*Cleome viscosa*	10	0.3	3	3.03	1.29	1.71	6.03
4	*Tridax procumbens*	10	1.1	11	3.03	4.76	6.28	14.07
5	*Euphorbia hirta*	12.5	1.52	12.2	3.78	6.58	6.96	17.32
6	*Portulaca oleracea*	5	0.07	1.5	1.51	0.30	0.85	2.66
7	*Hyptis suaveolens*	2.5	0.05	2	0.75	0.21	1.14	2.1
8	*Cyperus rotundus*	37.5	1.22	9.8	11.36	5.28	5.59	22.23
9	*Catharanthus roseus*	7.5	0.25	3.33	2.27	1.08	1.90	5.25
10	*Grangea maderaspatana* L.	12.5	1.85	14.8	3.78	8.00	8.45	20.23
11	*Sphaeranthus indicus*	15	1.25	8.33	4.54	5.41	4.75	14.7
12	*Barleria prionitis*	5	0.15	3	1.51	0.64	1.71	3.86
13	*Peperomia pellucida*	5	0.07	1.5	1.51	0.30	0.85	2.66
14	*Setaria verticillata*	7.5	0.62	8.33	2.27	2.68	4.75	9.7
15	*Hygrophylla auriculata*	12.5	0.57	4.6	3.78	2.46	2.62	8.86
16	*Tephrosia* sp.	7.5	0.27	3.66	2.27	1.16	2.09	5.52
17	*Ocimum* sp.	10	0.67	6.75	3.03	2.90	3.85	9.78
18	*Argemone mexicana*	10	0.5	5	3.03	2.16	2.85	8.04
19	*Solanum xanthocarpum*	20	0.5	2.5	6.06	2.16	1.42	9.64
20	*Acmispon brachycarpus*	7.5	0.3	4	2.27	1.29	2.28	5.84
21	*Parthenium hysterophorus*	40	4.1	10.25	12.12	17.74	5.85	35.71
22	*Achranthes aspera*	5	0.17	3.5	1.51	0.73	1.99	4.23
23	*Eleusina indica*	7.5	1.32	17.66	2.27	5.71	10.08	18.06
24	*Clarkia amoena*	7.5	0.22	3	2.27	0.95	1.71	4.93
25	*Lysimachia nummularia*	7.5	0.37	5	2.27	1.60	2.85	6.72
26	*Dichondra argentea*	5	0.1	2	1.51	0.4	1.14	3.05
27	*Jatropha curcas*	5	0.07	1.5	1.51	0.30	0.85	2.66
28	*Celosia*	5	0.32	6.5	1.51	1.38	3.71	6.6
	Total-	330	23.1	175.04	99.81	99.84	99.83	299.56
	Mean and standard deviation-	11.78 ± 98.15	0.825 ± 1.07	6.25 ± 19.0	3.56 ± 70.91	3.69 ± 20.08	3.56 ± 6.19	

**Table 3:** Properties of soil and heavy metal concentrations.

	GGV (Site- I)	NTPC(Site-II)	Mean and Standard Deviation
pH	7.0	6.7 (non-saline)	6.8 ± 0.4
Electrical conductivity	0.6 dS/m	0.4 dS/m	0.5 ± 0.01
Specific gravity	2.75	2.30	2.52 ± 0.1
Organic carbon	11%	0.45 %	
Nitrogen(N)	270 kg/ha	163 kg/ha	
Phosphorus(P)	20 kg/ha	11.64 kg/ha	
Potassium(K)	218.65 kg/ha	212 kg/ha	
Sulfur(S)	14 kg/ha	13.75 kg/ha	
Zinc(Zn)	9.8 kg/ha	0.412 kg/ha	
Boron(B)	2.0 kg/ha	5.0	
Iron (Fe)	15.12 mg/ha	39.46 mg/ha	
Manganese (Mn)	40 mg/ha	42.13 mg/ha	
Copper (Cu)	1.87 µg/g	2.003 µg/g	
Lead (Pb)	Non-detectable.	3.001 µg/g	

## Data Availability

All relevant data are included in the article.
